# The Role of Biochemical and Respiratory Markers in the Mortality of Patients With SARS-CoV-2 Infection in a Mexican Population

**DOI:** 10.7759/cureus.26249

**Published:** 2022-06-23

**Authors:** Jeshua Altuve-Quiroz, Carla Fernández-Reynoso, Michel G Mondragón-Soto, José I Juárez-Ramírez

**Affiliations:** 1 Department of Internal Medicine, Facultad Mexicana de Medicina, Universidad La Salle, Mexico City, MEX; 2 Department of Internal Medicine, Sanatorio Durango, Mexico City, MEX; 3 Department of Neurosurgery, Instituto Nacional de Neurología y Neurocirugía "Manuel Velasco Suárez", Mexico City, MEX

**Keywords:** hemato-immunological biomarkers, mortality, covid-19, biochemical markers, ventilatory support, sars-cov-2

## Abstract

Background: The SARS-CoV-2 pandemic has challenged the traditional perspectives of health care. The objective of our study was to analyze the association of different hematological biomarkers and respiratory assistance with the disease's severity and mortality in COVID-19.

Materials and methods: A single reference center, cross-sectional, retrospective, descriptive and analytical, observational study was carried out on 362 SARS-CoV-2 positive adults from April to October 2020.

Results: The mean age of the population was 55.92±13.12 years. A distribution by gender of n=227 (63.0%) men and n=135 women (37.0%) was found. Mortality occurred in 14% of the studied population. Comorbidities associated were hypertension n=128 (35.0%) and diabetes n=112 (31.0%). Of the 362 patients, 64 required advanced ventilatory support when taken to the intensive care unit, of these 39 (60.9%) died and only 25 (39.1%) survived (p<0.0001). On the other hand, biochemical indicators such as CRP, D-dimer, DHL, lymphocytes, leukocytes, neutrophils, and the neutrophil/lymphocyte ratio, showed a significant difference (p<0.0001) at admission and during the stay in the intensive care unit.

Conclusions: Patients who required ventilatory assistance showed an increased risk of mortality, as did those who were admitted to the intensive care unit. Higher mortality was associated with higher values ​​of CRP, DHL, D-dimer, neutrophil/lymphocytes ratio, total leukocytes, and lower lymphocytes.

## Introduction

The coronavirus disease 2019 (COVID-19) has been a global epidemic that caused extreme distress for the last 2 years through every possible aspect of the contemporary lifestyle and has challenged the known perspectives of healthcare [1]. Due to its high infection and mortality rate, and its association with underlying comorbidities, the public health system was overloaded [2].

Worldwide, the mortality reported ending December 31, 2021, totaled 5.94 million, it is estimated that the real count may be higher than three times as many people due to direct and indirect effects [3]. The total deaths reported in Mexico count up to 324,617 being the fifth place respecting the total number of casualties held [4]. The countries with the most excess deaths were India, the US, and Russia, followed by Mexico, Brazil, Indonesia, and Pakistan, in that order. Among these countries, excess mortality rates were highest in Russia and Mexico [3]. The cost of human lives and the economic burden are inestimable.

Although the epidemiologic data have shown a slowdown in virus transmission in certain parts of the world that were once deeply affected [5], the situation of Mexico has been harsh, as the application of COVID-19 vaccines has been delayed due to diverse factors, with a total of over 42 million people vaccinated [4]. This problem is associated, amongst others, with the high prevalence of chronic degenerative comorbidities [6]; however, there are studies where SARS-CoV-2 infection impact directly on biochemical markers such as the CRP, procalcitonin, TLC, lymphocyte count, D-dimer, as well as the appearance of thrombocytopenia. This suggested that biomarkers could be used to deepen the assessment elaborated on our patients for the prediction of the disease's outcome. On the other hand, currently, there is no consensus among medical societies regarding unified values from the biochemical perspective and their relationship with the severity of the disease.

In Italy and China, the first epicenters of the disease, different studies suggested that the proinflammatory state secondary to obesity was associated with the severity of the disease, independent of the patients' age. Until April 2020, there was no known relationship between inflammatory markers [1,7-10].

Among the diagnostic criteria that have been proposed, the most frequently reported have been lymphopenia, elevated procalcitonin, elevated IL-6, elevated IL-1β, low albumin, elevated creatinine, elevated urea nitrogen, D-dimer, prolonged prothrombin, and partial thromboplastin times. A consensus on absolute criteria for diagnosis and prognosis has not been homogenously established [11-13]. On the other hand, high liver enzymes have not shown a statistical difference despite the finding of the association between hepatic failure and COVID-19 [1].

Inflammatory biomarkers also have been reported to play a critical role as for every 1 mg/dL rise in C-reactive protein, 10 IU rise in lactate dehydrogenase (LDH), and 100 ng/mL rise in ferritin, the risk for moderate to severe disease was seen to rise by approximately 18%,13%, and 9%, respectively [14].

Information regarding the sensitivity and specificity of laboratory tests in determining severity of in the Mexican population has been reported before, but the size of the populations across the different studies is limited, thus, it provides an area of opportunity for finding reliable biomarkers that permit the assessment of the disease’s severity and prognosis in a timely fashion and allows the healthcare providers to adequately design a tailored plan of management and resource allocation [5], and to perform am adequate assessment of the changes in their values over time.

The present manuscript describes the association of biochemical markers and the incidence of mortality in patients with COVID-19, as well as the incidence of the different comorbidities reported and the functional outcome of our patients. After, we determined the diagnostic and prognostic performance of the different risk factors associated with the severity and mortality of the disease.

## Materials and methods

The present study was conducted in a single-center, retrospectively designed observation study, during the months of April through December 2020, when the second wave was at its peak in the region. A total of 362 patients were included, who were 18 years or older and diagnosed with COVID-19 using polymerase chain reaction (PCR) positive via nasopharyngeal or oropharyngeal swab. The patients were monitored for their disease course and outcomes. The study was approved by the Institutional Bioethics committee. The manuscript conformed to STROCCS guidelines for reporting cohort studies (Guidelines). Due to the retrospective nature of the study, the informed consent was waived.

The statistical analysis was conducted using the Statistical Package for Social Sciences (SPSS version 22.0, IBM Corp, Armonk, NY). Descriptive qualitative variables were presented as percentages and quantitative variables as means and standard deviation.

The initial Kolmogorov-Smirnov test was performed to find normal and non-normal distribution. Comparison of numerical variables between groups was performed using the Mann-Whitney U test or Student's t-test as required. All statistical tests with a p-value of <0.05 were considered statistically significant. A receiver operating characteristic analysis was also obtained to determine the predictive biochemical parameters for the outcome of death.

## Results

The mean age of our population was 55.92±13.12 years old, with 63% of the patients being males (n=227) and 37% were females (n=135) The rest of the demographic data are described in Table 1. Three hundred eleven patients were discharged to home, while 51 patients died (14%) while receiving in-hospital medical care secondary to SARS-CoV-2 infection.

**Table 1:** Differences between the biochemical values between deceased and alive patients diagnosed with COVID-19. Abbreviations: BMI-Body Mass Index

Variable	Deceased	Alive	P-value
BMI (kg/m^2^)	28.73 ± 5.01	29.55 ± 4.92	0.287
D-Dimer admission (ng/mL)	1226.47 ± 1176.60	1299.82 ± 1685.18	0.702
Procalcitonin admission (ng/mL)	1.52 ± 3.64	1.14 ± 8.89	0.604
Procalcitonin discharge (ng/mL)	12.19 ± 36.15	6.68 ± 67.61	0.401
Ferritin admission (ng/mL)	1100.95 ± 944.85	944.12 ± 1378	0.330

The incidence of comorbidities was reported to be 35% with hypertension, 31% of patients with diabetes mellitus, followed by other less frequent conditions such as chronic renal disease with 11.9% of patients. Hepatopathies were present in 1.7% of the patients.

There were differences in mortality when comparing age, oxygen saturation at the time of admission, CPR at admission and discharge, D-dimer at the time of discharge, ferritin at discharge, LDH at admission and discharge, total lymphocytes, total leucocytes, neutrophils, and NLR (Table 2). There were no outliers in the data, as assessed by inspection of a boxplot. The mortality was reported to be higher in elder patients, patients with lower saturation at the time of admission, higher CPR at admission and at the time of discharge, D-dimer at the time of discharge, higher ferritin at the time of discharge, higher LDH at the time of admission and discharge, higher leukocyte and neutrophil count lower lymphocyte count and higher neutrophil/lymphocyte count.

**Table 2:** Comparison of age, oxygen saturation, and laboratory values between alive and deceased patients diagnosed with COVID-19. Abbreviations: CRP - C-Reactive Protein, LDH - Lactate Dehydrogenase, NPL - Neutrophil-to-Lymphocyte Ratio

Variable	Deceased	Alive	P-value
Age (years)	66.80 ± 11.83	54.14 ± 12.46	≤ 0.0001
O2 saturation at admission (%)	80.27 ± 16.25	87.24 ± 6.46	≤ 0.0001
CRP at admission (ng/mL)	224.35 ± 151.11	135.76 ± 128.58	≤ 0.0001
CRP at discharge (ng/mL)	210.10 ± 182.80	32.94 ± 54.76	≤ 0.0001
D-Dimer at discharge (ng/mL)	5167.39 ± 4534.20	923.79 ± 1209.38	≤ 0.0001
Ferritin at discharge (μg/ml)	3348.48 ± 12179.53	789.04 ± 875.92	0.002
LDH at admission (UI/L)	410.04 ± 141.86	340.85 ± 138.71	0.001
LDH at discharge (UI/L)	742.59 ± 616.39	261.77 ± 89.06	≤ 0.0001
Total leucocyte count (cells/mm^3^)	8491.37 ± 4189.30	7311.48 ± 3236.28	0.022
Total Lymphocytes (cells/mm^3^)	700.49 ± 427.97	1008.23 ± 668.60	≤ 0.0001
Neutrophil count (cells/mm^3^)	7142.20 ± 4350.64	5893.83 ± 3102.54	0.013
NLR	14.09 ± 15.41	8.38 ± 9.40	≤ 0.0001

Of the 362 patient cohorts, 64 patients required ventilatory support after requiring admission into the Intensive Care Unit. Of these patients, 60.9% perished, and 39.1% survived. An independent-samples t-test was run to determine if there were differences in disease severity with biochemical markers. It was remarkable that only procalcitonin at the moment of admission and discharge showed no statistical differences, while higher CPR, LDH at discharge, lymphocytes, neutrophil count, neutrophil/lymphocyte, as well as lower LDH at admission, D-dimer at admission, leucocyte and lymphocyte count did (Table 3).

**Table 3:** Differences between hematological values in relation with the severity of disease and care at the ICU. Abbreviations: ICU - Intensive Care Unit, CRP - C-Reactive Protein, LDH - Lactate Dehydrogenase, NPL - Neutrophil-to-Lymphocyte Ratio

Variable	Moderate/severe	ICU/Very severe	P-value
CRP at admission (ng/mL)	135.08 ± 127.87	210.45 ± 148.33	< 0.0001
CRP at discharge(ng/mL)	31.37 ± 48.10	182.11 ± 182.72	< 0.0001
D-Dimer at admission (ng/mL)	1255.51 ± 1580.01	1447.92 ± 1804.49	0.434
D-Dimer at discharge (ng/mL)	9373.25 ±1389.76	4194.29 ± 2941.96	< 0.0001
Procalcitonin at admission	0.58 ± 3.67	4.10 ± 18.11	0.002
Procalcitonin at discharge	6.85 ± 69.04	10.34 ± 32.76	0.555
Ferritin at admission	902.71 ± 1114.36	1249.60 ± 2005.32	0.196
Ferritin at discharge	1177.04 ± 5511.30	1248.22 ± 1190.34	0.863
LDH at admission (UI/L)	418.87 ± 177.75	336.07 ± 127.61	< 0.0001
LDH at discharge (UI/L)	275.91 ± 146.18	555.41 ± 557.20	< 0.0001
Total leucocyte count at admission	8604.06 ± 4109.30	7235.55 ± 3190.11	0.003
Lymphocyte count and admission	1014.26 ± 680.74	734.93 ± 401.24	< 0.0001
Neutrophil count at admission	7279.11 ± 4228.65	5809.97 ± 3047.56	0.001
NLR at admission	8.32 ± 9.52	13.18 ± 14.11	0.001

Regarding the individual comorbidities, only hypertension and early CRD were associated with a higher risk of mortality (2.131 CI 1.172-3.875, p<0.05 and 2.39, CI 95% 1.120-5.132, p<0.05, respectively) (Table 4).

**Table 4:** Risk analysis of comorbities in relation to mortality in patients with COVID-19.

	Deceased	Alive	Odd’s ratio	P-value
Early Chronic Renal Disease				
Present	11 (25.6%)	32 (74.4%)	2.398 (IC95%:1.120 – 5.132)	0.010
Absent	40 (12.5%)	279 (87.5%)		
Systemic Arterial Hypertension				
Present	26 (20.3%)	102 (79.7%)	2.131 (IC95%: 1.172 – 3.875)	0.024
Absent	25 (10.7%)	209 (89.3%)		

Concerning the use of ventilatory support, there was increased mortality when either non-invasive or invasive mechanical ventilation or with their admission to the Intensive Care Unit. (OR 40.69, CI 95% 18.769-88.24, p<0.001, OR 57.682, CI 95% 25.51-130.429, p<0.001 and OR 37.18, CI 95% 17.296-79.925, p<0.001, respectively) (Table 5).

**Table 5:** Risk analysis of modalities of ventilation and ICU care in relation to mortality in patients with COVID-19. Abbreviations: ICU - Intensive Care Unit

	Deceased	Alive	Odd’s ratio	P-value
Non-invasive high flow ventilation				
Present	39 (62.9%)	23 (37.1%)	40.69 (IC95%: 18.769 – 88.240)	≤ 0.0001
Absent	12 (4.0%)	288 (96.0%)		
Intubated patients				
Present	38 (71.7%)	15 (28.3%)	57.682 (IC95%:25.510-130.429)	≤ 0.0001
Absent	13 (4.2%)	296 (95.8%)		
ICU admission				
Present	39 (60.9%)	25 (39.1%)	37.180 (IC95%: 17.296-79.925)	≤ 0.0001
Absent	12 (4.0%)	286 (96.0%)		

Higher TLC, Lymphocytes, were noticed in non-survivors among hematological indices. The inflammatory biomarkers including CRP, DHL, and DD were markedly increased in non-survived patients (p<0.001). Liver function enzymes were not discriminative among either group.

**Figure 1:**
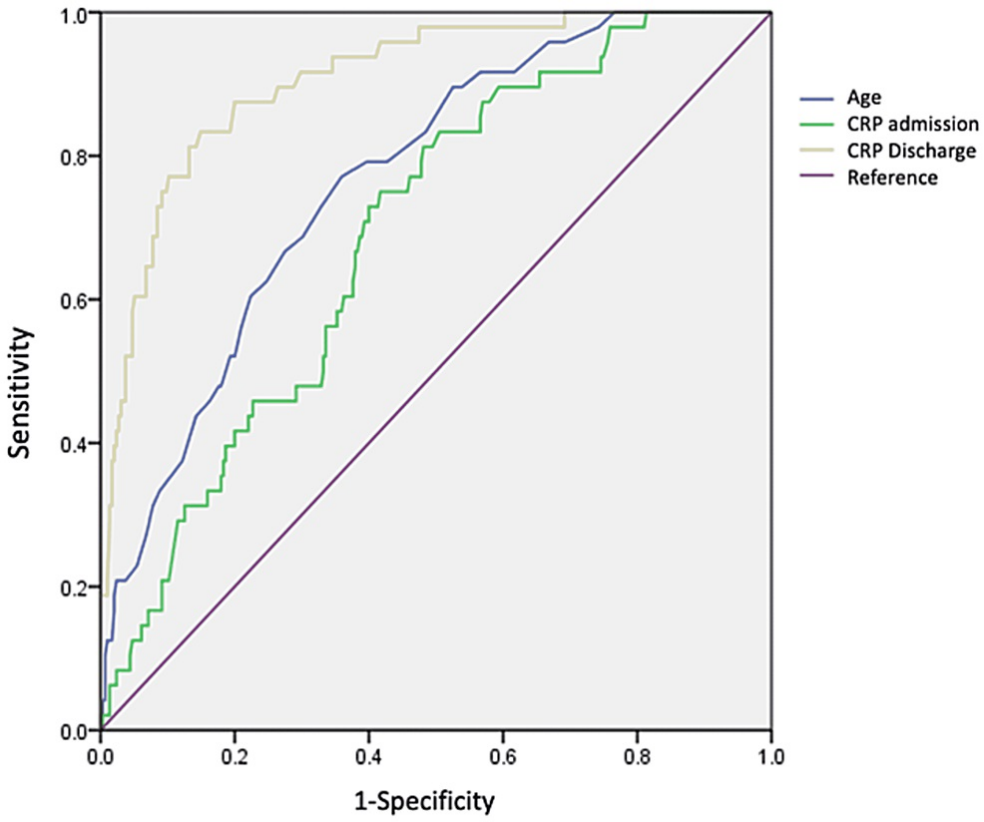
ROC curve for age, CRP at admission and discharge. Abbreviations: CRP - C-Reactive Protein

**Figure 2:**
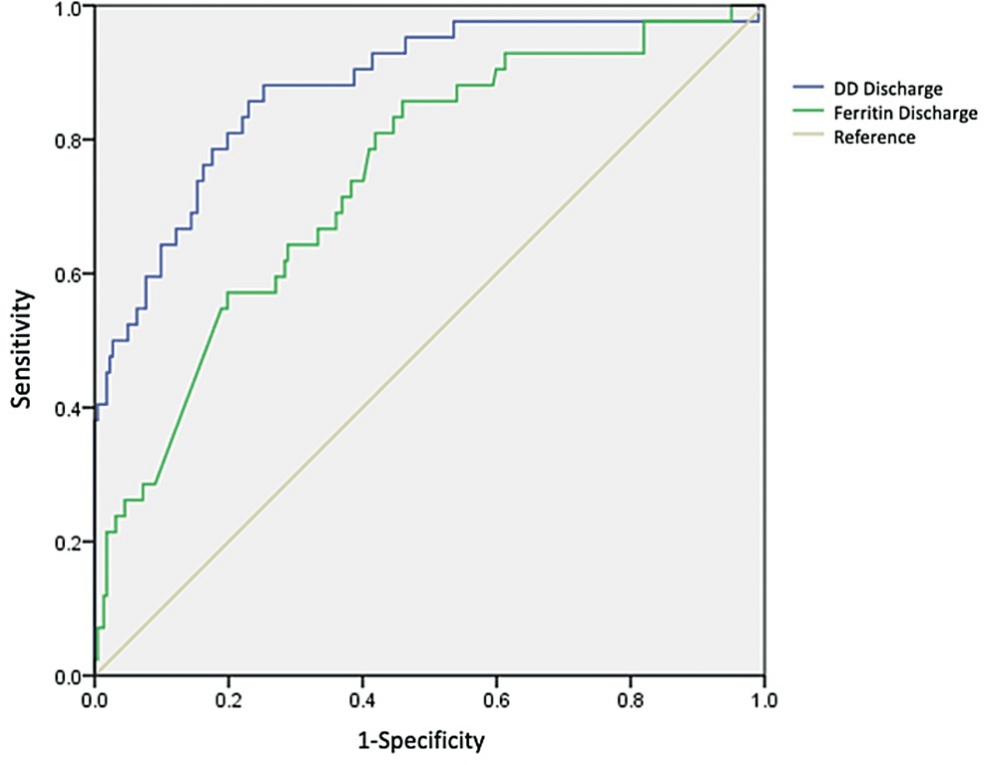
ROC curve for DD at admission and ferritin at admission. Abbreviations: ROC - Receiver Operating Characteristic, DD - D-Dimer

**Figure 3:**
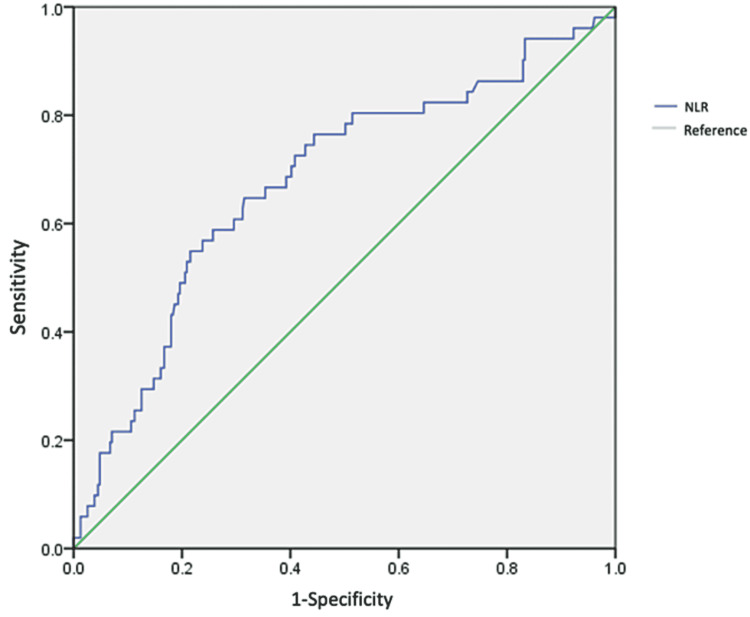
ROC curve for NLR. Abbreviations: ROC - Receiver Operating Characteristic, NLR - Neutrophil-to-Lymphocyte Ratio

On receiver operating curve (ROC) analysis, age had a cut-off point of 54.5 years old (AUC: 0.768) with a specificity of 0.83 and sensibility of 0.485, while inflammatory markers like CRP at discharge with a cut-off value of 48.5 (AUC:0.907, CI 0.864-0.950 p<0.0001) (Figure 1), ferritin with a cut-off value of 48.5(AUC: 0.744, IC95%: 0.664-0.824 p≤0.0001) DD at discharge with a cut-off point of 1,131.50 ngL/dL (AUC: 0.877, IC95%: 0.816-0.939 p≤0.0001) (Figure 2) and NLR with a cut-off point of 5.43 with a sensitivity of 51% and specificity of 80.4% (AUC : 0.804, IC95%: 0.594-0.761, p≤0.0001) were associated with elevated mortality (Figure 3).

## Discussion

COVID-19 is characterized by causing severe respiratory distress and has been associated with systemic inflammation that generates an important deterioration in the infected patients. It generates altered biochemical indicators which indicate impaired recovery mechanisms, which can lead to a mortality rate of 2% to 5% [10]. This host’s excessive inflammatory immunological response to SARS-CoV-2 infection has been hypothesized to be a major factor in disease severity and mortality. Several immune cells have been documented to be involved to play an essential role in the mechanism of the immune response regulation [15].

A systematic review on serum biomarkers for prediction of COVID-19 outcome included 1584 patients in their pooled analysis and found that the biomarkers that were noted to be significantly higher in those patients who died from coronavirus disease included: WBC, neutrophil count, CRP, high sensitivity C-reactive protein, procalcitonin, ferritin, D-dimer, interleukin-6, LDH, creatine kinase, prothrombin time, aspartate aminotransferase, alanine aminotransferase, total bilirubin, and creatine. Lymphocyte count, platelet count, and albumin were significantly lower in patients who died. The results from our study are comparative when taking into account the acute inflammatory biomarkers found in our cohort, but they differ when compared with liver function markers, where we found no statistically significant difference [5].

Differences in clinical presentation and outcomes in patients with COVID-19 are associated with racial/ethnic differences [16]. Arshad et al. published the association between increased mortality and severe disease in a population of patients with COVID-19 in Pakistan associated with different biomarkers with remarkable sensitivity and specificity, including CRP (86.36%/88.89%), ferritin (95.45%/86.57%), and LDH (90.91%/80.56%) [17]. In another study, increased blood urea nitrogen, elevated LDH, elevated NLR, elevated CRP, decreased albumin, and decreased natremia, which then led to the creation of a laboratory risk score for the early prediction of COVID-19 severity and in-hospital mortality, with a satisfying ability for the early prediction of both severity (AUC = 0.95) and mortality (AUC = 0.84), although this study's limitations include the retrospective single-center nature which may limit the generalizability of the findings and the short follow-up period comprehended in the report [18]. A meta-analysis reported higher mortality in patients with elevated white blood cell count, neutrophil count, CRP, procalcitonin, ferritin, D-dimer, IL-6, LDH, creatine kinase, prothrombin time, aspartate aminotransferase, alanine aminotransferase, total bilirubin, and creatine. Lymphocyte count, platelet count, and albumin [5]. These findings were similar to the ones encountered in our study, but the populations included did not comprehend a Latino population.

Concerning the Latin-American population, high fatality rates have been observed in Hispanics Specifically in Mexican patients, there have been some attempts to define the factors associated with COVID-19 severity and mortality. The presence of obesity, diabetes, hypertension, and CKD increases the mortality risk of COVID-19 [2,19,20], the mortality risk sharply increases with two or more comorbid diseases [19]. The Mexican population is at high risk of developing severe COVID-19 due to the high prevalence of overweight, with 70% of the population being overweight [21]. Cervantes et al. [16] performed a study of Hispanic 82 patients with COVID-19 on the USA-Mexico border, where he sought to evaluate the factors associated with COVID-19 severity and mortality where they reported hypertension (48.8%) and DM (39%), as well as advanced age, fever, and low SpO2, but no other biochemical value.

Other studies reinforce the association of biochemical markers and increased COVID-19 mortality in the Latino population, Ríos et al. published that the Leukocyte-to-Lymphocyte ratio, NLR, platelet-to-lymphocyte ratio, and nutritional index all were mortality predictors for COVID-19 [21]. Rosa et al. [2] found that non-survivors had significantly increased CRP, neutrophil count, NLR, ferritin, and AST compared to survivors. During the hospital stay, mortality was higher in patients who maintained elevated CRP, ferritin, neutrophil count, and NLR during the hospital stay, establishing a time-dependent relationship between the parameters studied and the prediction of mortality.

Another characteristic shared by patients with severe COVID-19 is the pulmonary involvement with the need for either non-invasive or invasive mechanical ventilation, where different findings have been reported, while limited diffusion may be responsible secondary to a severe inflammatory response, inspiratory airflow resistance has been documented to not to correlate with biomarkers of disease severity, and did not predict mortality [22]. Although higher mortality was found among the patients who received any modality of mechanical ventilation, different factors could confound these results, including the days of hospital stay, synchronous nosocomial infections, sepsis, barotrauma, multifactorial kidney injury, heart dysfunction, among others.

Different methods have been tested to determine COVID-19 prognosis, from cardiac biomarkers, echocardiogram findings, liver function tests, lacrimal, urine, and serum interleukins, to the detection of the molecular profile to identify the patient's inflammatory status using machine and deep learning [15]. Although the diagnostic and prognostic performance varies, the most available methods throughout the world include serology tests that can be performed virtually at any healthcare center, simplifying the disease's management and resource allocation, to do an adequate triage of the patients in a pandemic crisis setting.

The main limitation of the present study was the limited number of patients, which could probably be associated with not finding statistical significant differences among the rest of the variables considered, as well as the retrospective transversal nature of our study and the population pertaining to a single third level healthcare center.

## Conclusions

In this cohort study, we explore the usefulness of serologic biomarkers and the need for ventilatory support as prognostic indicators of mortality in Mexican patients with COVID-19. Patients who required ventilatory assistance showed an increased risk of mortality, as did those who were admitted to the intensive care unit. Higher mortality was associated with higher values ​​of CRP, DHL, D-dimer, neutrophil/lymphocytes ratio, total leukocytes, and lower lymphocytes. The identification and evaluation of these parameters could aid physicians to improve resource designation, avert the progression, and diminish the mortality of COVID-19. Further research is needed to correlate the mortality and the different biochemical markers, and their relevance in the clinical setting for the follow-up of patients with COVID-19.
